# A Proton Leak Current through the Cardiac Sodium Channel Is Linked to Mixed Arrhythmia and the Dilated Cardiomyopathy Phenotype

**DOI:** 10.1371/journal.pone.0038331

**Published:** 2012-05-31

**Authors:** Pascal Gosselin-Badaroudine, Dagmar I. Keller, Hai Huang, Valérie Pouliot, Aurélien Chatelier, Stefan Osswald, Marijke Brink, Mohamed Chahine

**Affiliations:** 1 Laval University Robert-Giffard Research Centre, Quebec City, Quebec, Canada; 2 Cardiology Department, University Hospital Zurich, Zurich, Switzerland; 3 Cardiology Department, University Hospital Basel, Basel, Switzerland; 4 Cardiobiology Research Laboratories, University Hospital Basel, Basel, Switzerland; 5 Department of Medicine, Laval University, Quebec City, Quebec, Canada; Leibniz-Institute for Arteriosclerosis Research at the University Muenster, Germany

## Abstract

Cardiac Na^+^ channels encoded by the *SCN5A* gene are essential for initiating heart beats and maintaining a regular heart rhythm. Mutations in these channels have recently been associated with atrial fibrillation, ventricular arrhythmias, conduction disorders, and dilated cardiomyopathy (DCM).

We investigated a young male patient with a mixed phenotype composed of documented conduction disorder, atrial flutter, and ventricular tachycardia associated with DCM. Further family screening revealed DCM in the patient's mother and sister and in three of the mother's sisters. Because of the complex clinical phenotypes, we screened *SCN5A* and identified a novel mutation, R219H, which is located on a highly conserved region on the fourth helix of the voltage sensor domain of Na_v_1.5. Three family members with DCM carried the R219H mutation.

The wild-type (WT) and mutant Na^+^ channels were expressed in a heterologous expression system, and intracellular pH (pHi) was measured using a pH-sensitive electrode. The biophysical characterization of the mutant channel revealed an unexpected selective proton leak with no effect on its biophysical properties. The H^+^ leak through the mutated Na_v_1.5 channel was not related to the Na^+^ permeation pathway but occurred through an alternative pore, most probably a proton wire on the voltage sensor domain.

We propose that acidification of cardiac myocytes and/or downstream events may cause the DCM phenotype and other electrical problems in affected family members. The identification of this clinically significant H^+^ leak may lead to the development of more targeted treatments.

## Introduction

The *SCN5A* gene codes for the α subunit of the human cardiac voltage-gated Na^+^ channel (Na_v_1.5) [1]. The most common phenotypes of *SCN5A* mutations are long QT syndrome type 3 (LQT3) [2] and Brugada syndrome (BrS) [3], [4], which can lead to malignant ventricular arrhythmias and sudden death [5]. Both syndromes are diagnosed on surface ECGs. The various clinical and ECG phenotypes of LQT3 and BrS arise from biophysical abnormalities of cardiac Na^+^ channel function. In general, LQT3 is caused by a gain of channel function while BrS is caused by a loss of channel function [6], [7]. Distinct cardiac phenotypes caused by *SCN5A* mutations have been described, including atrial fibrillation [8], sick sinus syndrome (SSS), conduction disorders such as atriventricular (AV)-block, and several more complex phenotypes [9]–[11], Dilated cardiomyopathy (DCM) is characterized by dilated cardiac chambers and reduced systolic function, which causes congestive heart failure. Patients with a family history of DCM account for approximately 20 to 25% of idiopathic DCM cases [12], [13].

Since it was first linked to the *SCN5A* gene in 1996 [14], DCM has been shown to be caused by a number of *SCN5A* mutations, including two frameshift mutations [15], a deletion mutation [16], and six missense mutations (T220I [17], R225W [18], R814W [15], A1180V [19], D1275N [15], [20], and D1595H [15]). With the exception of R814W, the other mutations have been associated with complex clinical phenotypes, including SSS, AV-block, and atrial and ventricular arrhythmias, as well as with divergent biophysical alterations of Na_v_1.5 [21]. Although, all these mutations are located on the voltage sensor [22], it is unclear which molecular mechanism is involved in the pathogenesis of DCM. Very recently, the well-known H558R polymorphism and alternative splice variant (Q1077del) were implicated in conduction system disease and DCM phenotypes in patients carrying the R222Q mutation in the *SCN5A* gene [23].

In the present study, we investigated a patient with a severe mixed phenotype who presented with cardiac conduction disorder and DCM. We identified a novel Na_v_1.5 mutation (R219H) that causes a proton leak through an alternative pathway unrelated to the Na^+^ pore.

## Methods

### Clinical evaluation

A detailed clinical history, a 12-lead ECG, a transthoracic ECG, and electrophysiological results were obtained at the initial assessment of the index patient. At the follow-ups, repetitive ECGs, Holter ECGs, echocardiograms, and stress-exercise tests were performed. Detailed clinical histories, 12-lead ECGs, and echocardiograms were obtained from the other family members.

### Molecular genetics

The index patient and family members provided written informed consent to participate in the study protocol, which was approved by the ethics committee of the University Hospital of Basel, Basel, Switzerland. Genomic DNA was extracted from whole blood samples. All *SCN5A* coding exons were amplified by polymerase chain reaction (PCR) using primers designed with intronic flanking sequences [24]. Denaturing high performance liquid chromatography (DHPLC) was performed on the DNA amplification products using at least one temperature condition. Products with abnormal DHPLC profiles were sequenced on both strands of the exon using a big dye termination mix and an automated laser fluorescent DNA sequencer (ABI Prism 377, Applied Biosystems). PCR and direct sequencing were used to identify the specific mutation in the family members. The mutation was absent in 200 control chromosomes. The sequence for the proximal connexin40 (Cx40) promoter in exon 1 carries GenBank accession number AF246295. Polymorphisms in the sequences upstream from Cx40 were genotyped by direct sequencing of PCR products generated using the forward primer 5′-TGAGGACAAGGACAACAGGCAG-3′ and the reverse primer 5′-CCTTCCTCTGGCTACTTCATATC-3′. The sequence for the coding region of Cx40 in exon 2 carries GenBank accession number AF151979. PCR products generated with the forward primer 5′-TGGAATCCCAGAACATGATAGA-3′ and the reverse primer 5′-TCAGTTCAGAAGGGAACAGTCT-3′ were directly sequenced.

### Na^+^channel mutagenesis

We used a cDNA construct encoding the human Na_v_1.5 Na^+^ channel [1]. Mutations were generated using QuikChange TM site-directed mutagenesis kits according to the manufacturer's instructions (Stratagene). Oligonucleotide primers containing the corresponding mutations were synthesized using the following sequences:

### For Na_v_1.5/R219H

5′-C AAT GTC TCA GCC TTA C**A**C ACC TTC CGA GTC CTC-3′ (forward primer)

5′- GAG GAC TCG GAA GGT G**T**G TAA GGC TGA GAC ATT G-3′ (reverse primer)

*For Na_v_1.5/C373F*

5′-CCTGATGACGCAGGACT**T**CTGGGAGCGCCTCTAT C-3′ (forward primer)

5′-GATAGAGGCGCTCCCAG**A**AGTCCTGCGTCATCAGG-3′ (reverse primer)

*For Na_v_1.5/R219A*

5′-CAATGTCTCAGCCTTAGCCACCTTCCGAGTCCTC-3′ (forward primer)

5′-GAGGACTCGGAAGGTG**GC**TAAGGCTGAGACATTG-3′ (reverse primer)

*For Na_v_1.5/R219C*

5′-CAATGTCTCAGCCTTA**T** GCACCTTCCGAGTCCTC-3′ (forward primer)

5′-GAGGACTCGGAAGGTGC**A**TAAGGCTGAGACATTG-3′ (forward primer)

*For Na_v_1.5/R219Q*

5′-CAATGTCTCAGCCTTAC**AG**ACCTTCCGAGTCCTC-3′ (forward primer)

5′-GAGGACTCGGAAGGTC**TG**TAAGGCTGAGACATTG-3′ (forward primer)

The codons are underlined, and the base changes are indicated in bold. The entire gene of each mutation was sequenced to check for undesirable mutations.

### Construct generation

Mutant and wild-type Na_v_1.5 Na^+^ channels were inserted in pPol1 [25], an oocyte expression vector containing theT7 promoter (5′ to 3′), the *Xenopuslaevis*β-globin 5′-untranslated region, a multiple cloning site, the *Xenopus laevis* β-globin 3′-untranslated region, a polyA tract, and a linearizing site. They were amplified in *E. coli* XL2 Blue (Stratagene) and purified using Genelute HP plasmid maxiprep kits (Sigma). The construct was linearized with *Not*I, and T7 RNA polymerase was used to make sense RNA using mMESSAGE mMACHINE T7 kits(Ambion).

### Xenopus oocytes

All experimental procedures involving Xenopus oocytes were approved by the Université Laval Institutional Animal Care Committee in line with the principles and guidelines of the Canadian Council on Animal Care (Approval 2011155-1), and were prepared as described previously [26]. Briefly, the oocytes were treated with 2 mg/ml of collagenase for 2 h. Stage IV or V oocytes were selected and were microinjected with capped mRNA coding for either the wild-type (WT) or mutant Na_v_1.5 channel (1 µg/µl of α-subunit and 0.3 µg/µl of β_1_-subunit, 50 nl/oocyte) or were mock injected with 50 nl/oocyte of sterile water. The oocytes were incubated at 18°C in oocyte recipe 3 (OR3) medium composed of a 1∶2 dilution of Leibovitz's L-15 medium (Invitrogene) supplemented with 15 mM 4-(2-hydroxyethyl)-1-piperazine-methanesulfonic acid (HEPES, pH 7.6, adjusted with NaOH), 1 mM glutamine, and 50 µg/µl of gentamycin. [26] They were used 3–4 days after injection. Macroscopic currents from the mRNA-injected oocytes were recorded using either the voltage-clamp technique with two 3 M KCl-filled microelectrodes or the cut-open oocyte technique [27]. The membrane potential for the two-microelectrode voltage-clamp technique was controlled using a Warner oocyte clamp (Warner Instrument Corp.). The currents were filtered at 2 kHz (−3 dB; 4-pole Bessel filter). For experiments using chloride-free solution, the headstage of the Warner amplifier was attached to a plastic pool containing a 3 M NaCl solution through a silver chloride wire connected to the bath solution using an agar bridge containing 3% agar, 500 mM N-methyl-D-glucamine (NMDG), and 10 mM HEPES (pH 7.4) and threaded with a thin platinum/iridium wire to increase electrical conductivity. The voltage for the cut-open oocyte technique was controlled using a CA-1B amplifier (Dagan Corporation) and mainframe clamp circuitry. The oocyte membrane was permeabilized using 0.1% saponin to provide low-resistance electrical access to the intracellular environment, perfuse the intracellular content, and remove chloride and potassium ions. Ca^2+^ was chelated with ethylene glycol tetraacetic acid (EGTA). Six agar bridges were used as electrical connections, and were fabricated as described above. Voltage commands were generated by computer using pCLAMP software version 10.0 (Molecular Devices). Currents were filtered at 5 kHz (−3 dB; 4-pole Bessel filter).

### pH_i_ measurements and electrode calibration

Intracellular pH (pH_i_) was measured using pH-sensitive electrodes. Briefly, borosilicate glass capillaries (Harvard Apparatus) were pulled using a Sutter Puller (Sutter Instruments Co.) and silanized with dichlorodimethylsilane (≥99.5%, Sigma) for 20 min in 5% (v/v) chloroform. After silanization, ≈0.5 µl of N_1_N_1_-dimethyltrimethylsilylamine proton exchange resin (Fluka) was placed in the tips of the capillaries using a 10-µl microsyringe (Hamilton). The capillaries were backfilled with Ringer's solution and were calibrated using different pH solutions before and after each experiment. The capillaries were then mounted on a holder with an Ag-AgCl pellet attached to the high-impedance amplifier of a two-channel FD223a electrometer (World Precision Instruments). Electrodes that did not match the specifications of the resin manufacturer (−57±1 mV/pH unit) were discarded. In some experiments where pH_i_ and proton currents were measured simultaneously under voltage clamp conditions, the oocytes were impaled with three electrodes, two standard microelectrodes for the voltage clamp and one containing the pH resin. A common reference electrode was used for both amplifiers.

### Solutions and reagents for Xenopus oocytes

The Ringer's bathing solution was composed of 116 mM NaCl, 2 mM KCl, 2 mM CaCl_2_, 2.9 mM MgCl_2_, and 5 mM HEPES. The pH was adjusted to 7.4 at 22°C using 1 M NaOH. The external chloride-free solution was composed of 120 mM N-methyl-D-glucamine (NMDG), 2 mM CaCl_2_, and either 20 mM TRIS (trishydroxyméthylaminométhane) (pH 8.4–8.6), 20 mM HEPES (4-(2-hydroxyethyl)-1-piperazineethanesulfonic acid)(pH 7.4 to 6.8), or 20 mM MES (2-(N-morpholino) ethanesulfonic acid) (<pH 6.8). The NMDG solutions were adjusted to the desired pH using methanesulfonic acid (Sigma). The intracellular NMDG solution was the same as the external solution for the cut-open oocyte technique except that the CaCl_2_ was replaced with 10 mM EGTA. All the recording solutions had an osmolarity of 240–260 mOsm. All the chemicals and drugs were purchased from Sigma except for tetrodotoxin, which was purchased from Latoxan (Valence, France). All the experiments were carried out at room temperature (≈22°C).

### Data analysis and statistics

The electrophysiological data was analyzed using macros in Campfit (pCLAMP v10.0, Molecular Devices) and custom programs written using MATLAB (The MathWorks Inc.). Statistical tests were performed using SigmaPlot 11(Systat Software Inc.). The results are expressed as means ± standard errors of the mean (SE). The number of measurements (n) is indicated in parentheses. Statistical comparisons were performed using an unpaired Student's *t*-test or Mann-Whitney's rank sum test. Differences were deemed significant at *p*<0.05. The *p* values are indicated in the text or figure legends.

## Results

### Clinical phenotypes of the index patient and the family members

**Figure 1:**
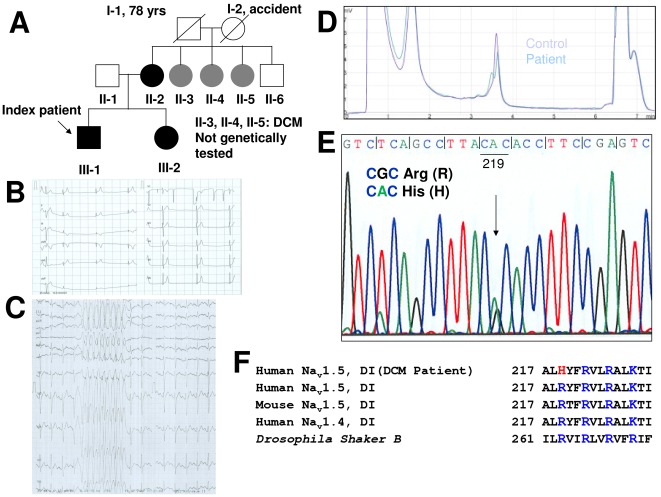
Family pedigree, clinical evaluation, and molecular genetics. (**A**) The index patient (III-1) is indicated by an arrow. Individuals indicated with black squares/circles carry the mutation and a clinical phenotype (III-1, III-2, II-2). Individuals indicated with grey circles (II-3 to II-5) were clinically diagnosed with DCM, but not genotyped. Abbreviation: DCM (dilated cardiomyopathy). (**B**) 12-lead ECG of the index patient showing third degree AV-block with a ventricular escape rhythm and a small QRS-complex with a heart rate of 43 bpm (artefact in lead V1). (**C**) Non-sustained ventricular tachycardia (220 bpm) occurred at a heart rate of 130 bpm and a work load of 192 W during an exercise stress test. (**D**) Different DHPLC eluting profiles at 59.8°C of the PCR products of exon 6 in the index patient compared to the control. Abbreviation: DHPLC (denaturing high performance liquid chromatography). (**E**) A heterozygous change of arginine CGC (R) to histidine CAC (H) resulted in the missense mutation R219H. (**F**) Sequence alignments of the S4 of domain 1 from Na^+^ and K^+^ (*Shaker* B) channels in different species.

The index patient was a 29-year old man in a three-generation family (**Figure 1A**), who presented with a four-day history of epigastric pain and dizziness. The clinical examination revealed bradycardia (43 bpm), and a 12-lead ECG showed a third degree AV-block with a ventricular escape rhythm, narrow QRS complex, and ventricular premature depolarizations (**Figure 1B**). Transthoracic echocardiography showed mild dilated cardiomyopathy with dilation of both atria and ventricles (left atrium = 43 mm, left ventricular end diastolic diameter (LVEDD) = 62 mm), with mildly decreased left ventricular ejection fraction (LVEF = 49%) and moderately decreased right ventricular systolic function (**see echocardiogram in Figure S1**). Acute coronary syndrome was excluded by repetitive negative troponins. A cardiac MRI revealed no signs of myocarditis or sarcoidosis and confirmed echocardiographic findings. A cardiac biopsy revealed unspecific findings. PCRs for common myocarditis infections were negative (e.g., enterovirus, parvovirus, herpes virus, Epstein Barr virus, adenovirus, cytomegalovirus, and *Borreliaburgdorferi*). A clinical electrophysiological study revealed a first degree AV block with intermittent third degree AV block, delayed A–H conduction (145 ms), a normal H-V-interval (45 ms), and no inducible ventricular tachycardia. A DDD pacemaker was implanted, and a combinational treatment with an ACE-inhibitor (perindopril) and indapamid was started. During a 3 monthsfollow up, the patient showed intermittent sinus rhythm whereas mild DCM was persistent with slightly decreased LVEF (52%). Eleven months later, during a bicycle exercise stress test (EST), the patient developed non-sustained ventricular tachycardia (220 bpm) at a heart rate of 130 bpm and a work load of 192 W (**Figure 1C**). A bisoprolol (10 mg/d) treatment was initiated. The follow-up EST with a maximal work load of 235 W and a maximum heart rate of 129 bpm showed no ventricular arrhythmias. Two months later, the patient was admitted to the hospital with atrial flutter (**Figure S2**). Immediate radiofrequency ablation of the atrial flutter was successfully performed. During further follow-up, the patient developed non-sustained ventricular tachycardia (Holter-ECG), and a cardioverter defibrillator was successfully implanted.

The family history revealed DCM in the patient's mother (II-2) and in three of the mother's sisters (**Figure 1A**). A transthoracic echocardiogram of the mother revealed a mildly dilated left atrium and a dilated left ventricle (LVEDD 57 mm), with mildly decreased systolic function (LVEF 48%). She suffered from frequent monomorphic premature ventricular contractions (PVC), but never revealed any episodes of atrial flutter, fibrillation or ventricular tachycardia. In the three sisters of the mother, echocardiography revealed the same echocardiographic phenotype with mild DCM; they all complained about infrequent PVC.

The older sister (III-2) of the index patient was diagnosed with borderline DCM, suffering from frequent PVC.

### Identification of a novel SCN5A mutation and family screening

DCM has been reported to be caused by a number of *SCN5A* mutations [21]. We identified a novel *SCN5A* mutation in the index patient. DHPLC (**Figure 1D**) and sequencing revealed a heterozygous change of CGC arginine (R) to CAC histidine (H), which resulted in the missense mutation R219H (**Figure 1E**). The family screening identified the mutation in the mother (II-2) and the patient's sister (III-2) (**Figure 1A**). The mother's three sisters with DCM (II-3, II-4, and II-5) refused genetic testing. The mutation was located in the S4 segment of the Na_v_1.5 voltage-sensor domain. This arginine residue is highly conserved in the voltage-sensor domain of ion channels in humans, squid (**Figure 1F**), and other species. Since it has been reported that an *SCN5A* DCM mutation (D1275N) can co-segregate with two connexin40 (Cx40) polymorphisms [20], [28], we sequenced the codons of these polymorphisms as well as the upstream regions and found no co-segregation with the R219H DCM mutation (**Figure S3**).

In addition, the H558R polymorphism is not present in the genotyped family members and the Q1077del splice variant was without effect on the proton current (data not shown).

### Na_v_1.5/R219H does not change the biophysical properties of Na^+^ channels expressed in Xenopus oocytes and tsA201 cells

**Figure 2:**
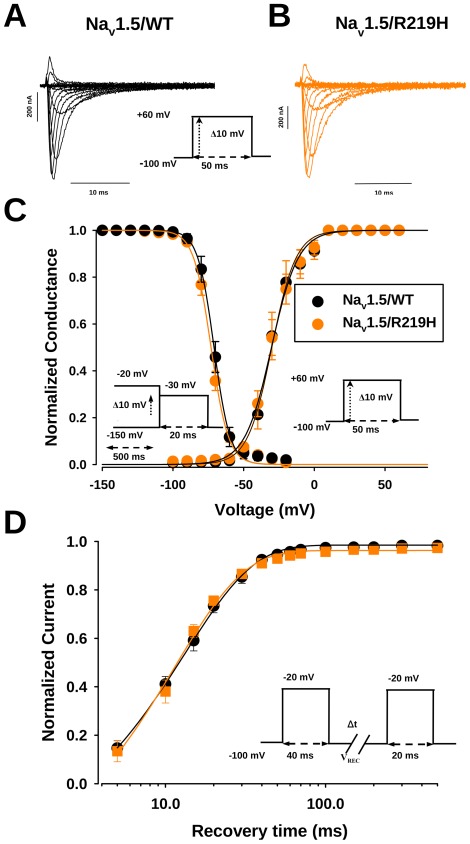
Biophysical characterization of the Na_v_1.5/R219H DCM mutation proton current recordings. Representative current traces recorded using the cut-open oocyte technique from Na_v_1.5/WT (**A**) and Na_v_1.5/R219H (**B**) channels. Currents were elicited by depolarizing pulses from −100 mV to +60 mV, with 10 mV increments for each step. (**C**) The voltage dependence of steady-state activation and inactivation of WT (activation, n = 7; inactivation, n = 8) and R219H (activation, n = 8; inactivation, n = 8). Activation curves were derived from *I*–*V* curves and fitted to a standard Boltzmann equation: *G* (*V*)/*G*_max_ = 1/(1+exp ((*V*−*V*_1/2_)/*k_v_*)), with midpoints (V_1/2_) is slow factors (*k_v_*) listed in **Table S1**. The voltage-dependence of inactivation was induced by applying conditioning pre-pulses to membrane potentials ranging from a holding potential of −150 to −20 mV for 500 ms with 10 mV increments and was then measured using a 20-ms test pulse to −30 mV for each step (see protocol in inset). The recorded inactivation data were fitted to a standard Boltzmann equation: *I* (*V*)/*I*_max_ = 1/(1+exp ((*V*−*V*_1/2_)/*k_v_*)), with midpoints (*V*_1/2_) is slow factors (*k_v_*) listed in **Table S1**. (**D**) Time courses of recovery from inactivation of Na_v_1.5/WT and Na_v_1.5/R219H channels. A 40 ms conditioning pre-pulse was used to monitor recovery using a 20-ms test pulse after a variable recovery interval ranging from 5 to 500 ms (see protocol in inset). A single-exponential function was used to determine the time constants of recovery.

Robust Na_v_1.5 Na^+^ currents were recorded from both WT and R219H mutant channels using the cut-open *Xenopus* oocyte system. They exhibited a typical pattern of rapid voltage-dependent activation and inactivation kinetics (**Figure 2A–B**). The peak currents for the WT and R219H channels were plotted as normalized conductance-voltage (G–V) curves (**Figure 2C**). No significant differences between the WT and R219H channels were observed. Steady-state inactivation showed no alterations in the best-fit parameters for both the slope and midpoint of inactivation (**Figure 2C** **and Table S1**). No significant effects were observed for recovery from fast inactivation (**Figure 2D** **and Table S1**). The patch clamp studies using the tsA201 mammalian expression system corroborated the *Xenopus* oocytes experiments (**Figure S4A–F and Table S2**). The methods for the patch clamp experiments could be found in Figure S4 legend.

### The R219H mutant Na^+^ channel conducts an inward pH-dependent current at hyperpolarizing voltages

The nature of the R to H amino acid substitution and its involvement in generating proton leaks in *Shaker* and Na_v_1.4 ion channels prompted us to verify whether the mutant channels leak H^+^ ions [29], [30]. We used the *Xenopus* oocyte system because it allows the expression of high levels of Na^+^ channel proteins. This system also circumvents many limitations of HEK293 mammalian cells such as the presence of ASIC channels that are sensitive to extracellular pH variations. [31]

**Figure 3:**
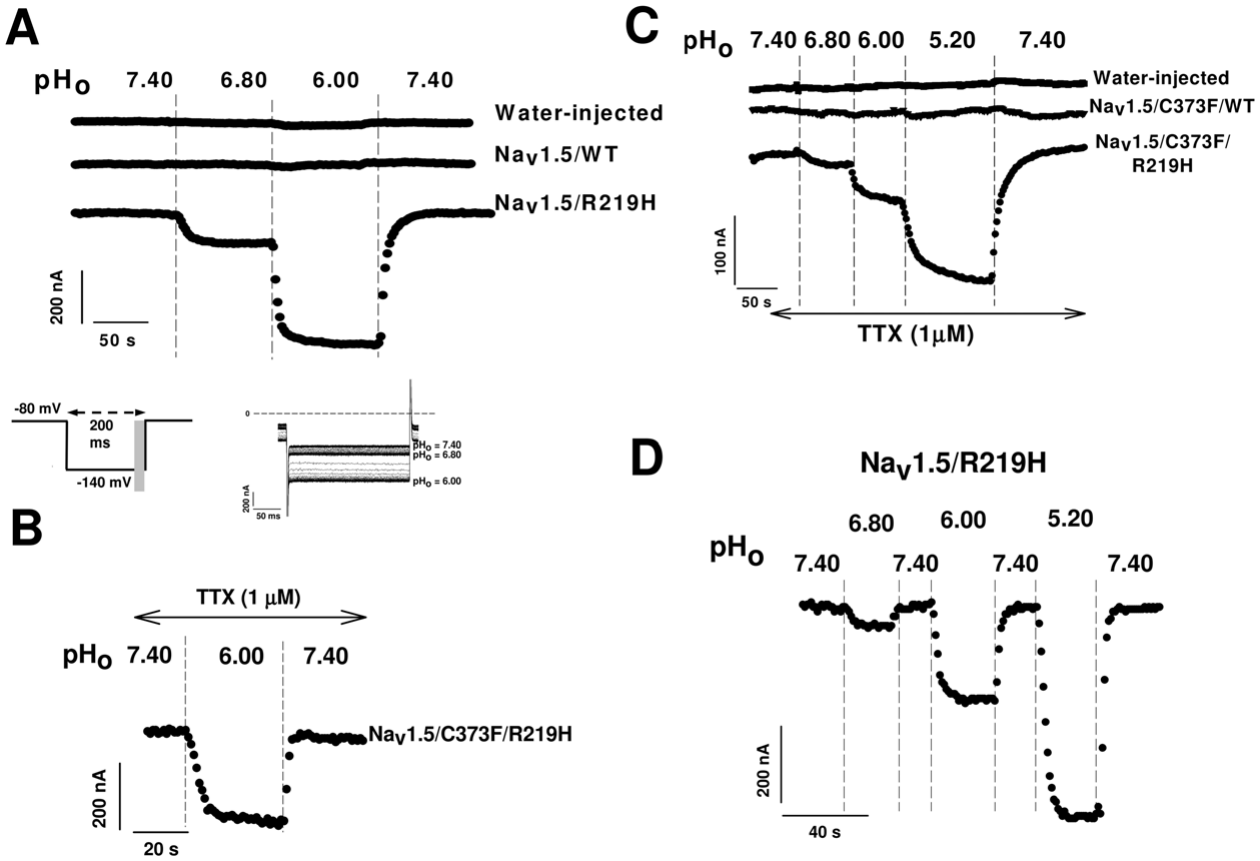
Na_v_1.5/R219H exhibits a pH dependent current. Panel (**A**) shows that varying the Ringer's extracellular pH (pH_o_) induced an inward current in Na_v_1.5/R219H–injected oocytes. An acidic Ringer's solution induced inward currents in an oocyte expressing the Na_v_1.5/R219H channel. The oocyte was held at −80 mV and a −140 mV test pulse was repeated every 2 s (only current responses at −140 mV are shown). The bottom panels show current traces of the experiment in (**A**). The inset shows the protocol, and the grey zone indicates where currents were measured as a mean of the current amplitude between 150 and 200 ms.(**B**) Effect of an acidic Ringer's solution in the presence of TTX in an oocyte expressing the Na_v_1.5/R219H mutant channel in a background in which the native cysteine in D1 had been replaced with a tyrosine (C373F) and in the presence of 1 µM TTX. This mutation in the pore region increases the TTX sensitivity of cardiac channels 60- to100-fold, as described previously [46]. (**C**) Currents recorded from a water-injected and oocyte expressing the Na_v_1.5/WT or Na_v_1.5/R219H channel. The oocytes were held at −80 mV and were pulsed to −140 mV. This protocol was repeated every 2 s as indicated in the inset (**panel A**). Acidic NMDG solutions induced a pH-dependent current in an oocyte expressing the Na_v_1.5/R219H channel in a C373F background in the presence of 1 µM TTX. This experiment was carried out in a Na_v_1.5 background in which the native cysteine in D1 was replaced with a tyrosine (C373F). (**D**) Acidic NMDG solutions induced a reversible current in an oocyte expressing the Na_v_1.5/R219H channel.

While subjecting the oocytes to a pulse protocol (**Figure 3A** **inset**), we changed the extracellular pH (pH_o_) and observed an increased inward current that was several hundred nA in amplitude when the pH_o_ of the Ringer's solution was reduced from 7.40 to 6.80 or lower for oocytes expressing the Na_v_1.5 mutant channel (**Figure 3A**). This pH-dependent inward current was not affected by TTX (1 µM), a selective pore blocker of voltage-gated Na^+^ channels (**Figure 3B**), indicating that the inward current pathway in mutant channels differed from that of the channel pore.

To distinguish this leak current from the alpha current (pore of the channel), Na^+^ ions were substituted by NMDG. No chloride ions were added in order to reduce chloride current contamination. In a Na^+^-free NMDG solution, mutant Na^+^ channels displayed a significantly increased inward current at pH_o_ 7.40 and lower (**Figure 3C**). However, an acidic external pH did not induce a current in WT-injected or water-injected oocytes (**Figure 3C** **top panels**). The inward current increased as the pH_o_ become more acidic, and the effect was reversible (**Figure 3c**). The pH-dependent inward current was fully reversible (**Figure 3D**). To elucidate the specific role of the histidine at position 219, we created a series of mutants by replacing R219 with alanine, glutamine, lysine, or cysteine. Acidic pH_o_ values did not induce an inward current in these mutants (**Figure S5A–D**).

### The pH-dependent inward current is a H^+^ current

**Figure 4:**
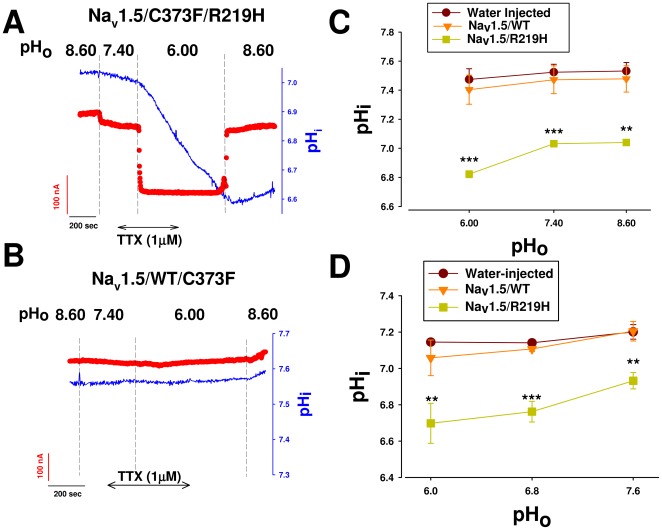
Na_v_1.5/R219H induces an inward proton current and intracellular acidification. *Xenopus* oocytes expressing Na_v_1.5/WT or Na_v_1.5/R219H channel were impaled with three electrodes, one filled with an H^+^ resin to measure pH_i_, and two to clamp the oocyte at −80 mV in a Na^+^-free NMDG solution containing 1 µM TTX, as indicated. Typical proton current recordings (red traces) in response to different pH_o_ value and the pH_i_ measurement rate (bleu traces) from an oocyte expressing the Na_v_1.5/R219H (**A**) or Na_v_1.5/WT channel (**B**). Intracellular pH_i_ values before changing solutions in experiments similar to (**A**) and (**B**) were plotted against pH_o_ (***, p<0.001 compared to WT, n = 10–19)(**C**). Similar recordings were obtained with four batches of oocytes. (**D**) Changes in pH_i_ after incubating oocytes expressing the Na_v_1.5/WT (triangles) or Na_v_1.5/R219H (squares) channel, or water-injected oocytes (circles) in OR3 medium at different pH_o_ values (***, p<0.001, **; p<0.01; *, p<0.05; compared to WT, n = 7–13). pH_i_ measurements were carried out in Ringer's solution at pH_o_ of 7.40.

We used an H^+^-selective electrode to directly measure intracellular pH and to test the hypotheses that this inward current is a proton current and that the protons conducted through the mutant channel can induce intracellular acidification. We simultaneously recorded currents using a two-microelectrode technique in Na^+^-free NMDG solution in the presence of 1 µM TTX as indicated in **Figure 4A**. Inward currents paralleled intracellular acidification, especially at lower pH_o_ (6.80–6.00) values. This suggested that the inward current observed is a proton current and that it causes intracellular acidification. This effect was not affected by 1 µM TTX and was partially reversible (**Figure 4A**). No current was detected and no acidification was observed in Na_v_1.5/WT-injected oocytes (**Figure 4B**). **Figure 4C** shows the degree of intracellular acidification at different pH_o_ values in oocytes expressing WT or R219H mutant channels and in water-injected oocytes.

We also measured the pH_i_ to determine whether proton currents flowing through the mutant channel contribute to intracellular acidification at rest. Oocytes expressing WT or R219H channels as well as water-injected oocytes were incubated in OR3 medium (see methods) for 18 h at different pH_o_ values and were transferred into the recording chamber. pH_i_ was measured using a pH-sensitive electrode. The incubation of the oocytes at acidic pH_o_ values resulted in significant intracellular acidification, unlike the slight acidification observed with oocytes expressing WT channels and with water-injected oocytes (**Figure 4D**). This suggested that there may be a flow of H^+^ ions into cardiac myocytes at the resting potential (−80 mV).

**Figure 5:**
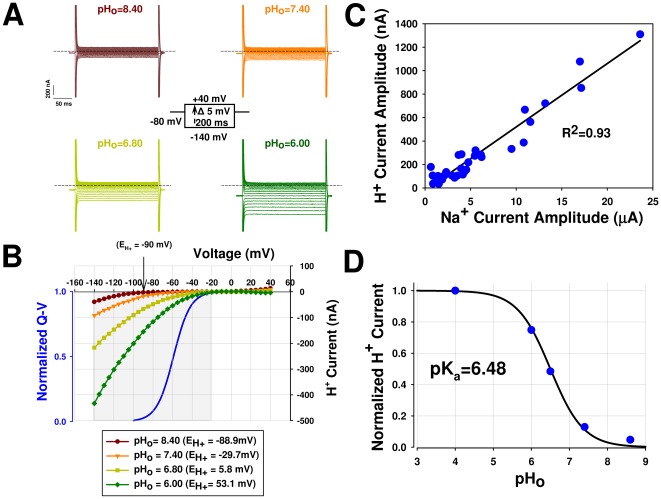
Proton current-voltage relationship of the Na_v_1.5/R219H channel recorded in an NMDG Na^+^-free solution. (**A**) Representative proton current traces from oocytes expressing the Na_v_1.5/R219H channel recorded at pH_o_ 8.40, 7.40, 6.80, and 6.00, as indicated, in response to 200 ms voltage steps ranging from −140 mV to +40 mV in 5-mV increments from a holding potential of −80 mV (the protocol is given in the centre inset), without on-line leak subtraction. The dashed line represents the zero current. For clarity, only current every 10 mV are shown. (**B**) Current-voltage relationship where the currents in (**A**) were plotted as a function of the test potential (5 mV increments), after offline linear leak subtraction. Reversal potential determined in a Na^+^-free NMDG solution at pH_o_ 8.40 using voltage steps as described in (**A**). The pH_i_ was measured using a pH-sensitive electrode. Similar results were obtained with four separate batches of oocytes. The inset shows the pH_o_and pH_i_ values and between parentheses is the predicted values calculated using the Nernst equation. The bleu trace shows the voltage-dependent of activation (Q–V), the grey zone illustrates the transitional zone corresponding to the probability of the voltage sensor being stabilized in the outward position. (**C**) Correlation between the peak Na^+^ current measured in Ringer's solution and the proton current measured at −140 mV and pH_o_ 4.00 (n = 31) on the same oocytes. The data were obtained from one batch of oocytes over three days. The straight line represents the linear regression of the data set and R^2^ is the correlation coefficient and shows the goodness of fit. Similar results were obtained with three separate batches of oocytes. (**D**) Proton currents measured in response to a change in pH_o_ at −140 mV in an NMDG Na^+^-free solution. The currents were normalized to the currents obtained at pH_o_ = 4.00 for each cell. The mean data (n = 5) was fitted to the Henderson-Hasselbach equation, 1/[1+exp(2.3(pH_o_−pK_a_))]. Error bars are smaller than the symbols.

The voltage-dependence of the proton current was recorded using 10 mV voltage steps from −140 mV to +40 mV from a holding potential of −80 mV (**Figure 5A**). The proton current was pH_o_-dependent, was higher at more hyperpolarizing voltages (**Figure 5A**), and exhibited a voltage-dependent inward rectification that occurred near −50 mV at pH_o_ 7.40 (**Figure 5B**). This is the voltage at which the voltage sensor (shown by measuring the Q–V curve representing the gating-charge movement), and thus the histidine, moves outward, disconnecting it from the H^+^ permeation pathway.

The reversal potential was measured to determine whether the observed current was selective for H^+^(**Figure 5B**). We measured the pH_i_ using a pH-sensitive electrode while recording inward currents. Voltage steps were applied from a holding potential of −80 mV, and ranged from −140 mV to +40 mV. To ensure that the reversal potential could be measured, a more basic extracellular pH (pH_o_8.40) was used, keeping in mind an important constraint that, at pH_o_<7.40, the expected reversal potential is more depolarized than −50 mV. More positive potentials than −50 mV reflect a situation in which S4 voltage sensors are predicted to be stabilized in an upward position, resulting in a decrease in the H^+^ current. These measurements were made with oocytes in which the expression of Na^+^ channels was very high and in which currents could be detected at pH_o_ 8.40. **Figure 5B** (filled circles) shows a reversal potential measured using pH-sensitive electrode in an oocyte expressing Na_v_1.5/R219H where the pH_i_ was 6.90. The observed reversal potential (≈−90 mV) was close to the predicted value calculated using the Nernst equation (E_H_ = RT/zF*ln [H_o_]/[H_i_] = −88.9 mV) and was consistent with a highly-selective H^+^ current.

The proton current increased in tandem with the expression of the Na^+^ channels with a significant correlation (R^2^ = 0.93) and made up 5% of the peak Na^+^ current (**Figure 5C**), suggesting that the α subunit was responsible for the proton current.

The histidine residue was titrated to confirm its involvement in generating proton currents. The titrated curve generated by plotting the pH_o_ values against the proton current amplitude measured at −140 mV fit well with the Henderson-Hasselbach equation (**Figure 5D**). The measured pK_a_ was 6.48±0.01 (n = 5), which was in agreement with the pK_a_ of the histidine in an aqueous solution (pK_a_ = 6.5).

## Discussion

### Clinical aspects of the DCM patient

Human molecular genetic studies have uncovered over 30 distinct genes linked to the pathophysiology of DCM [32], [33]. While it is known that *SCN5A* mutations are involved in ventricular arrhythmia, the same cannot be said for structural heart diseases such as DCM. Three DCM-affected family members were genotyped and were found to carry the R219H mutation. They showed a robust genotype-to-phenotype correlation, suggesting that the DCM was strongly associated with the R219H mutation. No co-segregation of R219H with two nucleotide polymorphisms inthe regulatory region of Cx40 (−44AA, +71GG), which has been reported to reduceCx40 expression levels that could contribute to the atrial electrical abnormalities, was found [28]. This suggests that R219H was the main mutation causing atrial electrical disturbances associated DCM. Additional DCM or arrhythmia genes were not screened for mutations as SCN5A was considered the target gene for the mixed cardiomyopathy-arrhythmia phenotype. The same mutation was reported in a Japanese family with Sick sinus syndrome [34]. For unknown reasons the mutation did not express in these authors hand. In our hand this mutation was fully functional.

### Biophysical properties of R219H channels

The basic biophysical properties (activation, inactivation, recovery from inactivation, and kinetics) of the mutated channels expressed in either *Xenopus* oocytes (Figure 2) or the tsA201 mammalian cell line (Figure S4) were not altered. Furthermore, no persistent Na^+^ current could be detected (data not shown).

While several Na_v_1.5 mutations have been identified in patients with DCM that may contribute to the disease, the molecular mechanisms underlying their involvement are poorly understood. It is difficult to explain such clinical phenotypes solely on changes in the electrical properties of cardiac sodium channels.

### The R219H mutation induces a pH-dependent inward current that is a H^+^ current

We tested the hypothesis that mutant channels may leak H^+^ and are thus sensitive to changes in extracellular pH. Titrating histidine at pH_o_ 7.40 and above resulted in a reversible inward H^+^ current at hyperpolarized voltages, in contrast to water-injected oocytes and WT Na_v_1.5-injected oocytes in which no current was generated. Furthermore, the H^+^ current was not affected by 1 µM TTX. These findings indicated that the inward H^+^ current is TTX-insensitive and, more importantly, that H^+^ protons do not leak into the cell through the Na^+^ ion permeation pathway but through an alternative pathway. In addition, the current was activated by hyperpolarizing voltages at which there is very little if any likelihood of the α pore opening.

We used H^+^-selective electrodes to determine whether the H^+^ protons conducted through the mutant channel induced intracellular acidification. We simultaneously recorded H^+^ currents using a two microelectrode technique in Na^+^-free condition. Inward H^+^ currents paralleled intracellular acidification, especially at more acidic pH_o_ values (6.80 and 6.00), suggesting that the inward current observed was indeed an H^+^ current that caused intracellular acidification

Similar H^+^ channels can be formed by replacing the most positively charged arginine residue of the *Drosophila Shaker* voltage-gated K^+^ channel with a histidine [29]. Recent studies have associated similar leak currents through the ω pore of Na_v_1.4, the skeletal muscle Na^+^ channel, with hypokalemic periodic paralysis [30], [35]. The extent to which this pore also allows H^+^ permeation requires further investigations.

Na_v_1.5/R219H generated a proton current, even in the presence of 300 µM amiloride at acidic pH values (data not shown), suggesting that amiloride-sensitive channels and H^+^ transporters do not contribute to this current and that amiloride does not inhibit this current. We tested several potential blockers, including Ni^2+^, Cd^2+^, Zn^2+^, La^3+^, and ethylguanidine, none of which inhibited this current at millimolar concentrations (data not shown).

### Proposed mechanisms for proton permeation

Replacing the arginine residue with a histidine revealed a permeation pathway across the voltage sensor domain of Na_v_1.5 Na^+^ channel, most probably *via* a proton wire route, through which H^+^ translocates into the cell in a way similar to what has been proposed for gramicidin-A channels and *Shaker* potassium channel [29], [36]. Proton conduction through gramicidin-A channels has been described as occurring *via* a hop-and-turn mechanism. This mechanism involves H^+^ ion hops between water molecules, which account for the high H^+^ selectivity observed. The proton current-voltage relationship of R219H exhibited strong voltage-dependent rectification (**Figure 5B**), which occurred near −50 mV (pH_o_ of 7.40). At more depolarized voltages, the voltage sensors are more stabilized in an outward position and thus disrupt the proton wire. The permeation pathway may thus be favoured by the inward position of the D1S4 helix at hyperpolarized voltages (<−50 mV) and may be blocked when the S4 helix moves outward (>0mV), with a transition zone between −50 and 0 mV in which the probability of this movement taking place increases (**Figure 5B** **, grey zone**), thus disrupting the proton wire [37], [38].

### Pathophysiology

A proton current through mutated cardiac Na^+^ channels can depolarize cardiac myocytes and make the resting membrane potential unstable and thus contribute to premature ventricular depolarizations. H^+^ leaking into cardiac myocytes may disrupt pH_i_ homeostasis and transform the proton leak into a Na^+^ leak, leading to intracellular Na^+^ accumulation *via* the activation of H^+^ transporters, including the Na^+^-H^+^ anti-port exchanger [39]. The increase in Na^+^ can lead to Ca^2+^ accumulation *via* the reverse mode of the Na^+^/Ca^2+^ exchanger [40], which may modify calcium homeostasis and lead to changes in the contractile properties of the myocyte [41]. The accumulation of Ca^2+^ may also have a deleterious effect and may contribute to DCM phenotypes. It is also likely that acidosis directly affects myofilament sensitivity to Ca^2+^, which has been reported previously to reduce the affinity of troponin C for Ca^2+^ and thus impair excitation-contraction coupling [42]. These ionic homeostatic alterations, including acidification, may also directly or indirectly (e.g., *via* phosphorylation status) uncouple gap junctions, as already suggested for Cx40 [43] and Cx43 [44]. Acidification is also deleterious for gap junction integrity [45]. The sensitivity of cardiac connexins to acidification probably contributes to the diverse conduction disturbances and arrhythmias seen in our patient. Future research using a cellular model (stem cells) or an animal model reproducing the R219H mutation (knock-in mice) is warranted.

Our study revealed an unexpected, but pathologically significant, proton current associated with the cardiac Na^+^ channel. This novel proton leak current is essentially a gain-of-function because it induces an H^+^ leak that is not present when arginine occupies position 219. It may thus cause DCM and complex electrical phenotypes. It is noteworthy that heart failure therapy with a beta-blocker and perindopril/indapamid controlled the progression of DCM in our patient. The ideal pharmacological strategy would be to inhibit the H^+^ leak without affecting normal channel gating and Na^+^ permeability.

## Supporting Information

Table S1 **Biophysical properties of Na_v_1.5/WT and Na_v_1.5/R219H obtained using the cut-open oocyte technique.**
(DOC)

Table S2 **Biophysical properties of Na_v_1.5/WT and Na_v_1.5/R219H obtained using the patch clamp technique.**
(DOC)

Figure S1 **Echocardiogram of the index patient's heart.** Apical four chamber view showing dilation of both atria and ventricles with mildly decreased left and moderate decreased right ventricular systolic function: LVEDD 59 mm, LVESD 50 mm, IVSd 10 mm, PWd 8 mm, LA 48 mm, LVEF 50%. EDV 236 ml, EDVI 118 ml/m2. LVEDD: left ventricular end-diastolic diameter; LVESD: left ventricular end-systolic diameter; IVSd: inter-ventricular septum diastolic; PWd: posterior wall diastolic; LA: left atrium, LVEF: left ventricular ejection fraction; EDV(I): End-diastolic left ventricular volume (Index).
(TIF)

Figure S2 **12-lead ECG showing atrial flutter with 3∶1 conduction on the index patient.**
(TIF)

Figure S3 **Connexin40 genotyping.** To investigate whether the Na_v_1.5/R219H could co-segregate with the already reported Cx40 polymorphisms^1^, we sequenced the entire coding region of Cx40 and Cx40 upstream sequences, in the mother the father and the two siblings. Although the mother and her son were phenotypically (DCM) and genotypically (R219H) similar, they differ in polymorphisms on Cx40 upstream sequences proposed to change Cx40 expression levels. The mother (**patient II-2**) was homozygote [−44AA (**a**), +71GG (**b**)), conditions where the expression of Cx40 is markedly reduced^1^]. However, the index patient (**patient III-1**) was heterozygote at both positions [−44AG and +71AG]. Groenewegen, W.A. *et al.* A cardiac sodium channel mutation cosegregates with a rare connexin40 genotype in familial atrial standstill. *Circ. Res.* **92**, 14–22 (2003).
(TIF)

Figure S4 **Biophysical characterization of Na_v_1.5/R219H Na^+^ channels expressed in tsA201 cells.** Representative current traces recorded from Na_v_1.5/WT (**a**) and Na_v_1.5/R219H (**b**). Currents were elicited by depolarizing pulses starting at −100 mV to +50 mV with a 10 mV increment for each step from a holding potential of −140 mV, as shown in the inset protocol. (**c**) Current-voltage relationship of WT and R219H. Current amplitude was normalized to the membrane capacitance to generate the corresponding current density. (**d**) The activated and inactivated currents were generated from the protocols as inset. Using the same data as (**c**) and graphically determined reversal potentials (Erev), the Na^+^ conductance (G) for the various voltages was calculated from the equation G = I/(V−Erev). The fraction of the conductance was obtained by normalizing the various conductances at different voltages to the top values. Steady-state inactivation was measured by applying 500 ms pre-pulses ranging from −140 to −50 mV, followed by a 20 ms test pulse at −30 mV. The resulting data of steady-state activation and inactivation were fitted to a standard Boltzmann distribution. (**e**) Slow inactivation in WT and R219H. A two-pulse protocol as inset was used to generate the currents. The course of slow inactivation was assessed using a two-pulse protocol with an initial conditioning pre-pulse and a final test pulse. A −30 mV pre-pulse was applied at intervals varying from 1 to 1000 ms, followed by a step to −140 mV for 20 ms to allow the channels to recover from fast inactivation. The −30 mV test pulse was applied for 40 ms to estimate the fraction of channels available for activation. Time constants (shown in Table S2) were obtained using a mono-exponential function. (**f**) Time courses of recovery from slow inactivation in WT and R219H. A 500 ms conditioning pre-pulse was used to monitor recovery by a 20 ms test pulse after a variable recovery interval from 1 to 1000 ms (see protocol in inset). A two-exponential function was used to obtain the resulting time constants (shown in Table S2). Results are presented as means ± standard error. *Methods* *Figure S4:* TsA201 cells were grown in high glucose Dulbecco's modified Eagle's medium (DMEM) supplemented with fetal bovine serum (10%), L-glutamine (2 mM), penicillin G (100 U/ml), and streptomycin (10 mg/ml) (Gibco). The cells were incubated in a 5% CO_2_ humidified atmosphere after being transfected with WT or mutant human Na_v_1.5 cDNA (2 µg) and human β_1_-subunit (2 µg) using the calcium-phosphate method. The human Na channel β_1_-subunit and CD8 were inserted in the pIRES bicistronic vector in the form of pCD8-IRES-β_1_. Using this strategy, transfected cells that bound beads also expressed the β_1_-subunit protein. Transfected cells were incubated in the medium containing anti-CD8-coated beads (Dynal) for 2 min before performing patch-clamp experiments. Cells expressing CD8 were distinguished from non-transfected cells by visualizing beads fixed on the cell membrane by light microscopy. *Sodium currents recordings:* The whole-cell configuration of the patch clamp technique was used to record macroscopic Na currents from transfected tsA201 cells. Patch clamp recordings were obtained using low-resistance, fire-polished electrodes (<1 MΩ) made from 8161 Corning borosilicate glass coated with Sylgard (Dow-Corning) to minimize electrode capacitance. Currents were recorded with an Axopatch 200 amplifier (Molecular), and series resistance was >80% compensated. Command pulses were generated, and currents were acquired using a Pentium-based computer running pCLAMP software v8.0 equipped with a DigiData 1300 AD converter (Molecular Devices). P/4 leak subtraction was used to compensate for linear leaks and eliminate capacitative transients. Currents were filtered at 5 kHz and digitized at 10 kHz. All recordings were performed at room temperature (22–23°C). Cells were permitted to stabilize for 10 min after establishing the whole-cell configuration before recording currents. A 7 mV junction potential between the patch electrode and the bath solution was corrected. *Solutions and reagents:* For the whole-cell recordings, the patch pipettes were filled with a solution containing 35 mM NaCl, 105 mM CsF, 10 mM EGTA, and 10 mM Cs-HEPES. The pH was adjusted to 7.4 using 1 N CsOH. The bath solution consisted of 150 mM NaCl, 2 mM KCl, 1.5 mM CaCl_2_, 1 mM MgCl_2_, 10 mM glucose, and 10 mM Na-HEPES. The pH was adjusted to pH 7.4 using 1 N NaOH (final Na^+^: 152.4 mM).
(TIF)

Figure S5 **Effect of alanine, glutamine, lysine and cysteine substitution.** The arginine 219 was substituted with alanine (**a**), glutamine (**b**), lysine (**c**) and cysteine (**d**), and oocytes expressing mutant channels were superfused with Na^+^-free NMDG solution at different pH_o_. Proton currents were measured every 2 seconds, using a hyperpolarizing pulse of −140 mV from a holding potential of −80 mV, as indicated in the inset. No proton currents could be seen in the presence of all mutant channels except for the cysteine mutant, where a slight inward deflection of the current at extreme acidic pH_o_ value (5.20) (d) was observed, but we did not study this effect in greater detail. Similar results were obtained in two separate batches of oocytes.
(TIF)
