# Correction: A Proton Leak Current through the Cardiac Sodium Channel Is Linked to Mixed Arrhythmia and the Dilated Cardiomyopathy Phenotype

**DOI:** 10.1371/annotation/03304989-b70c-400c-b752-2e3d18851047

**Published:** 2012-06-08

**Authors:** Pascal Gosselin-Badaroudine, Dagmar I. Keller, Hai Huang, Valérie Pouliot, Aurélien Chatelier, Stefan Osswald, Marijke Brink, Mohamed Chahine

The first two authors, Pascal Gosselin-Badaroudine and Dagmar I. Keller, should be noted as contributing equally to this work. 

