# Supplementary material for: A Proton Leak Current through the Cardiac Sodium Channel Is Linked to Mixed Arrhythmia and the Dilated Cardiomyopathy Phenotype
Source: PLoS One. 2012 May 31;7(5):e38331. doi: 10.1371/journal.pone.0038331 (PMC3365008; doi:10.1371/journal.pone.0038331)
Supplement: Table S1 — Biophysical properties of Nav1.5/WT and Nav1.5/R219H obtained using the cut-open oocyte technique. (DOC) [file pone.0038331.s001.doc]

**Table S1. Biophysical properties of Na_v_1.5/WT and Na_v_1.5/R219H obtained using the cut-open oocyte technique.**

|  | **Na_v_1.5/WT** | **Na_v_1.5/R219H** |
| --- | --- | --- |
| *Steady-state activation* |  |  |
| *V*_1/2_ (mV) | -29.15 ± 3.10 (n=6) | -30.85 ± 2.70 (n=9) |
| *k*_v_ | -6.22 ± 1.43 (n=6) | -5.30 ± 0.97 (n=9) |
| *Steady-state inactivation* |  |  |
| *V*_1/2_ (mV) | -75.14 ± 1.21 (n=7) | -71.47 ± 1.85 (n=7) |
| *k*_v_ | 5.94 ± 0.42 (n=7) | 5.54 ± 0.74 (n=7) |
| *Recovery from inactivation*  (-100mV) |  |  |
| *τ* _rec_ (ms) | 11.55 ± 0.57 (n=8) | 13.04± 1.85 (n=8) |
|  |  |  |

*V*_1/2_ = midpoint for activation or inactivation;

*k*_v_ = slow factor for activation or inactivation

*τ* _rec_ = time constant of recovery from inactivation;

n = number of measurements

Values are presented as means ± standard error. All Na_v_1.5/R219H parameters were not statistically different compared to Na_v_1.5/WT counterparts.
