# Supplementary material for: A Proton Leak Current through the Cardiac Sodium Channel Is Linked to Mixed Arrhythmia and the Dilated Cardiomyopathy Phenotype
Source: PLoS One. 2012 May 31;7(5):e38331. doi: 10.1371/journal.pone.0038331 (PMC3365008; doi:10.1371/journal.pone.0038331)
Supplement: Table S2 — Biophysical properties of Nav1.5/WT and Nav1.5/R219H obtained using the patch clamp technique. (DOC) [file pone.0038331.s002.doc]

**Table S2. Biophysical properties of Na_v_1.5/WT and Na_v_1.5/R219H obtained using the patch clamp technique.**

|  | **WT** | **R219H** |
| --- | --- | --- |
| ***Steady-state activation*** |  |  |
| ***V*_1/2_ (mV)** | -55.33 ± 0.86 (n=7) | -57.82 ± 0.65 (n=7) |
| ***k* (mV)** | -5.84 ± 0.31 (n=7) | -5.76 ± 0.23 (n=7) |
| ***Steady-state inactivation*** |  |  |
| ***V*_1/2_ (mV)** | -100.14 ± 0.77 (n=7) | -101.56 ± 0.54 (n=7) |
| ***k* (mV)** | 5.52 ± 0.24 (n=7) | 6.64 ± 0.23 (n=7) |
| ***C* (%)** | 0.16 ± 0.03 (n=7) | 0.98 ± 0.09 (n=7) |
| ***Slow inactivation*** |  |  |
| ***τ* (ms)** | 335.49 ± 54.36 (n=6) | 346.62 ± 32.87 (n=7) |
| ***Recovery from slow inactivation*** |  |  |
| ***τ* _fast_ (ms)** | 3.4 ± 0.35 (n=8) | 3.24 ± 0.23 (n=9) |
| ***τ* _slow_ (ms)** | 86.37 ± 9.56 (n=8) | 75.76 ± 10.34 (n=9) |
| ***A* _fast (%)_** | 96.09 ± 2.24 (n=8) | 95.11 ± 1.69 (n=9) |
| ***A* _slow (%)_** | 4.92 ± 2.03 (n=8) | 5.78 ± 1.87 (n=9) |

*V*_1/2_ = mid point for activation or inactivation;

*k*_v_ = slow factor for activation or inactivation

*C*= value of the persistent sodium currents in steady-state inactivation;

*τ* = time constant;

*A* = the fractions of recovery component

Values are presented as means ± standard error.
