# Supplementary figures and images for: A Proton Leak Current through the Cardiac Sodium Channel Is Linked to Mixed Arrhythmia and the Dilated Cardiomyopathy Phenotype
Source: PLoS One. 2012 May 31;7(5):e38331. doi: 10.1371/journal.pone.0038331 (PMC3365008; doi:10.1371/journal.pone.0038331)

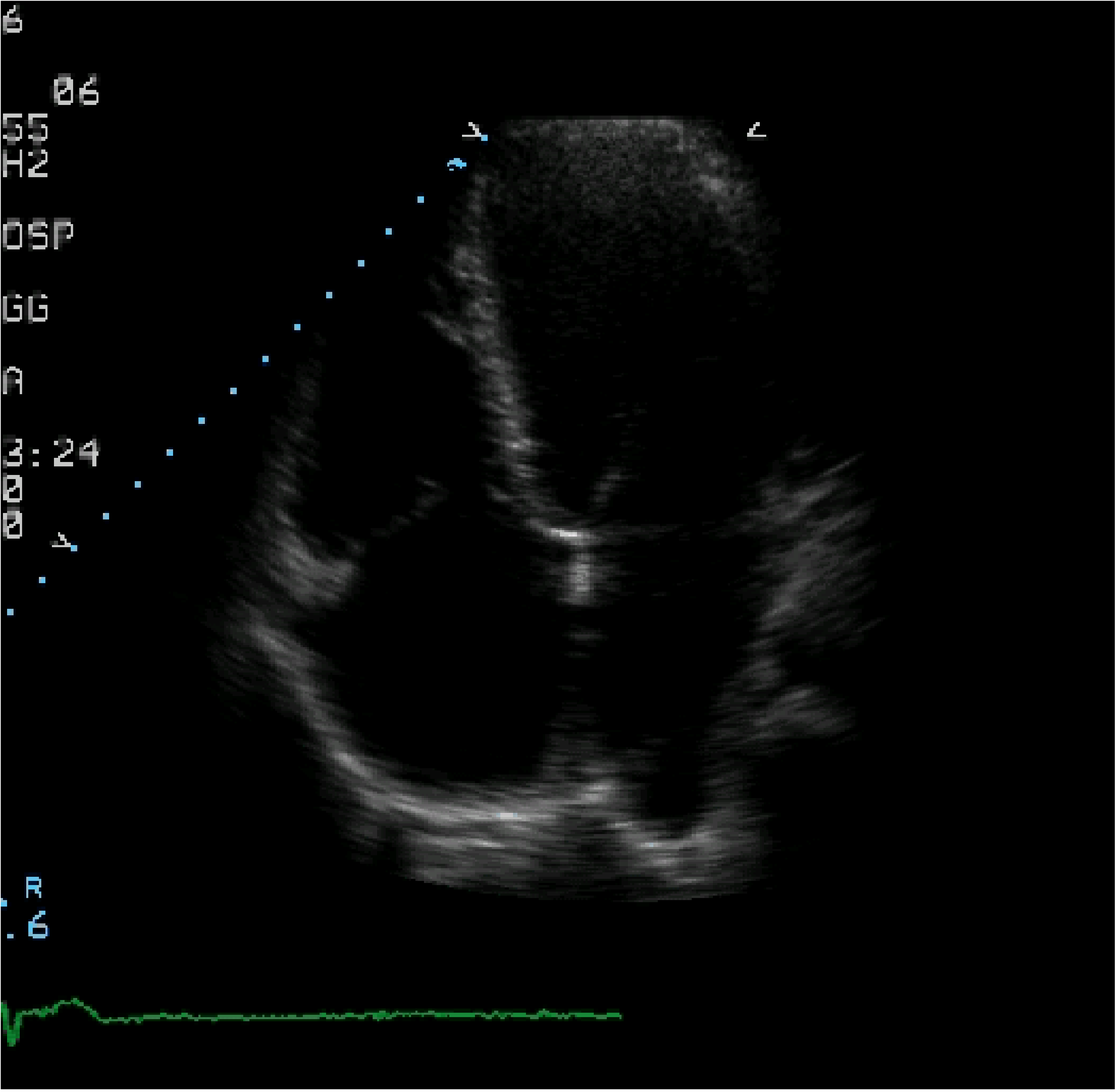

Supplement: Figure S1 — Echocardiogram of the index patient's heart. Apical four chamber view showing dilation of both atria and ventricles with mildly decreased left and moderate decreased right ventricular systolic function: LVEDD 59 mm, LVESD 50 mm, IVSd 10 mm, PWd 8 mm, LA 48 mm, LVEF 50%. EDV 236 ml, EDVI 118 ml/m2. LVEDD: left ventricular end-diastolic diameter; LVESD: left ventricular end-systolic diameter; IVSd: inter-ventricular septum diastolic; PWd: posterior wall diastolic; LA: left atrium, LVEF: left ventricular ejection fraction; EDV(I): End-diastolic left ventricular volume (Index). (TIF) [file pone.0038331.s003.tif]

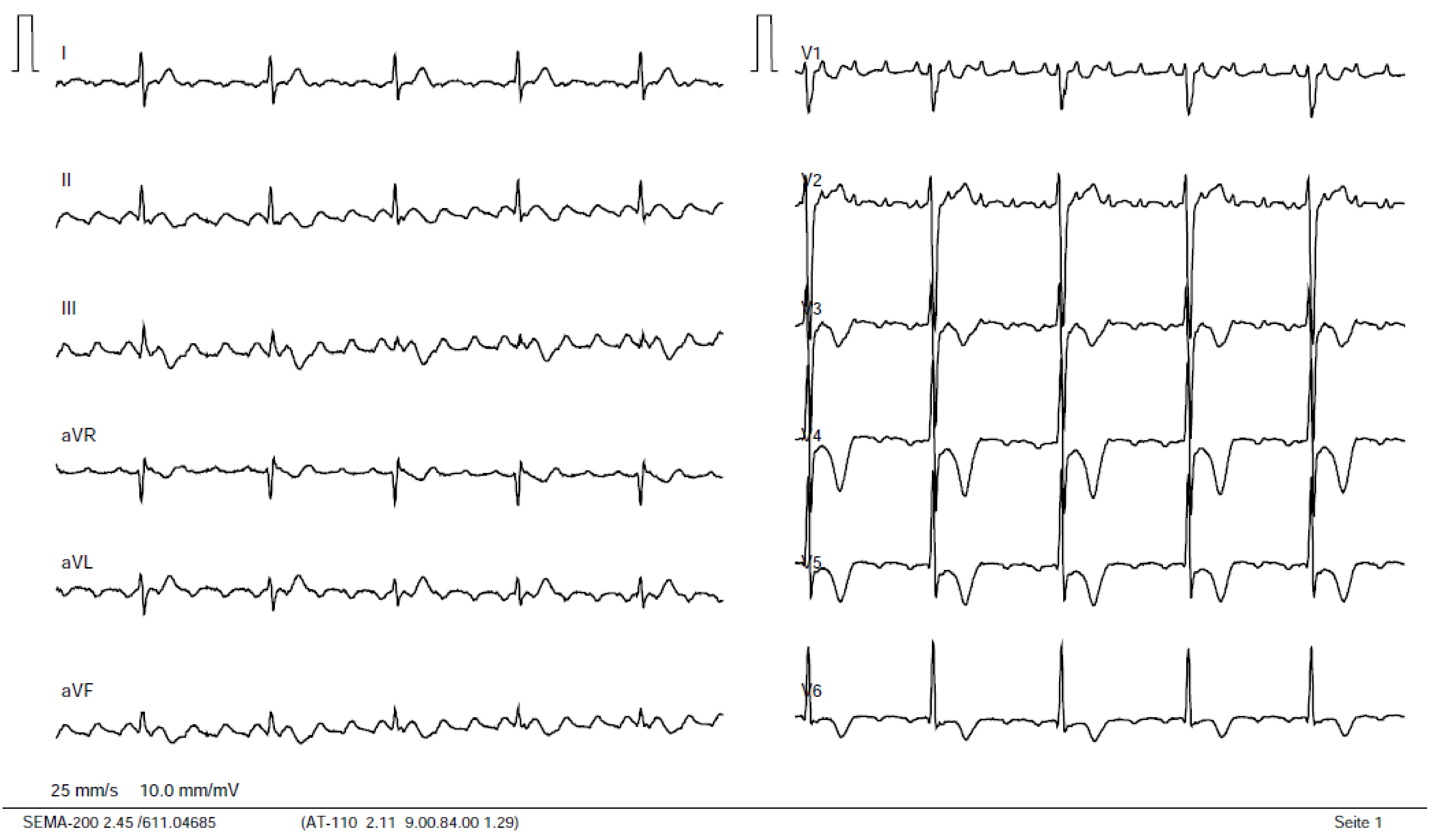

Supplement: Figure S2 — 12-lead ECG showing atrial flutter with 3∶1 conduction on the index patient. (TIF) [file pone.0038331.s004.tif]

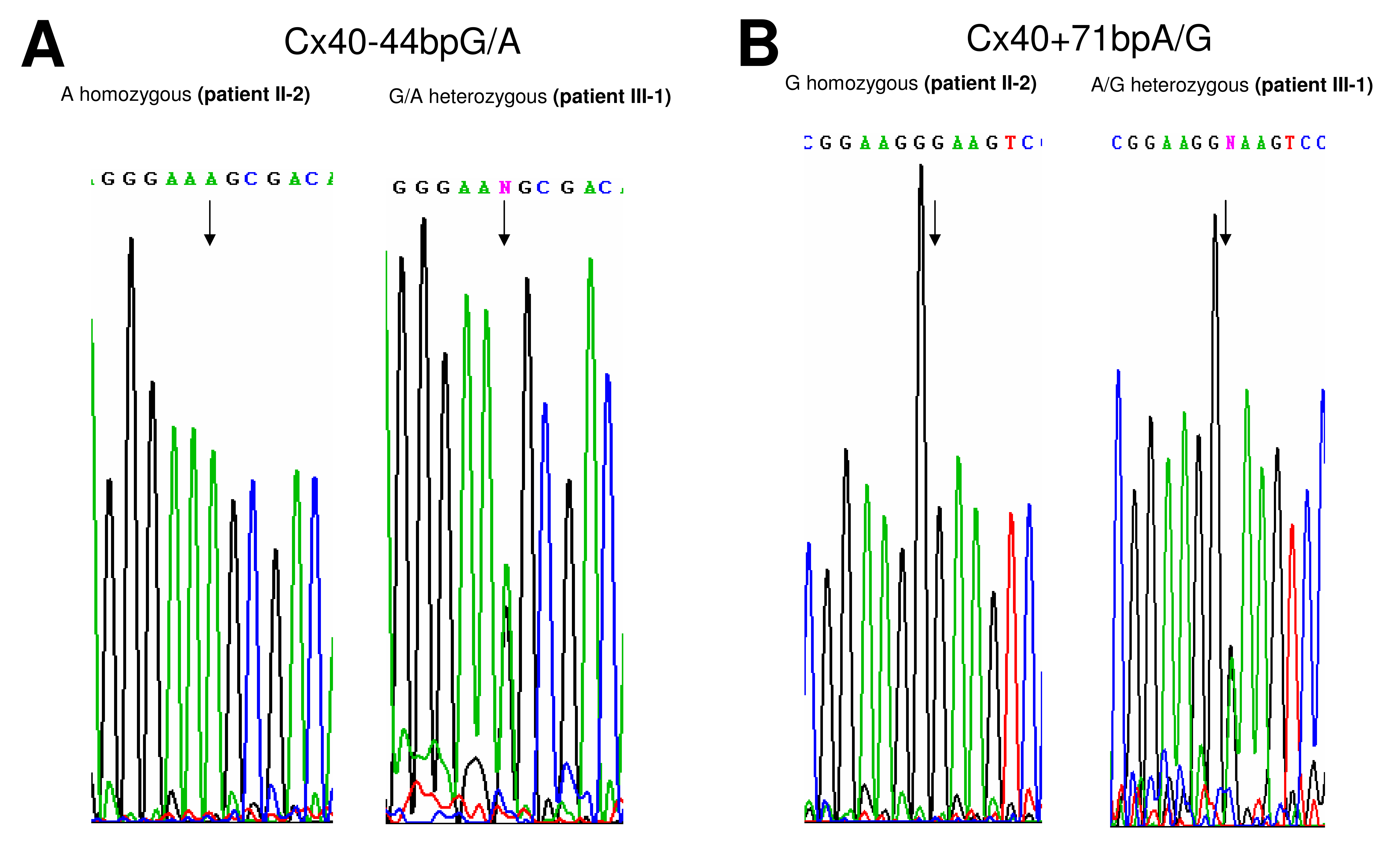

Supplement: Figure S3 — Connexin40 genotyping. To investigate whether the Nav1.5/R219H could co-segregate with the already reported Cx40 polymorphisms1, we sequenced the entire coding region of Cx40 and Cx40 upstream sequences, in the mother the father and the two siblings. Although the mother and her son were phenotypically (DCM) and genotypically (R219H) similar, they differ in polymorphisms on Cx40 upstream sequences proposed to change Cx40 expression levels. The mother (patient II-2) was homozygote [−44AA (a), +71GG (b)), conditions where the expression of Cx40 is markedly reduced1]. However, the index patient (patient III-1) was heterozygote at both positions [−44AG and +71AG]. Groenewegen, W.A. et al. A cardiac sodium channel mutation cosegregates with a rare connexin40 genotype in familial atrial standstill. Circ. Res. 92, 14–22 (2003). (TIF) [file pone.0038331.s005.tif]

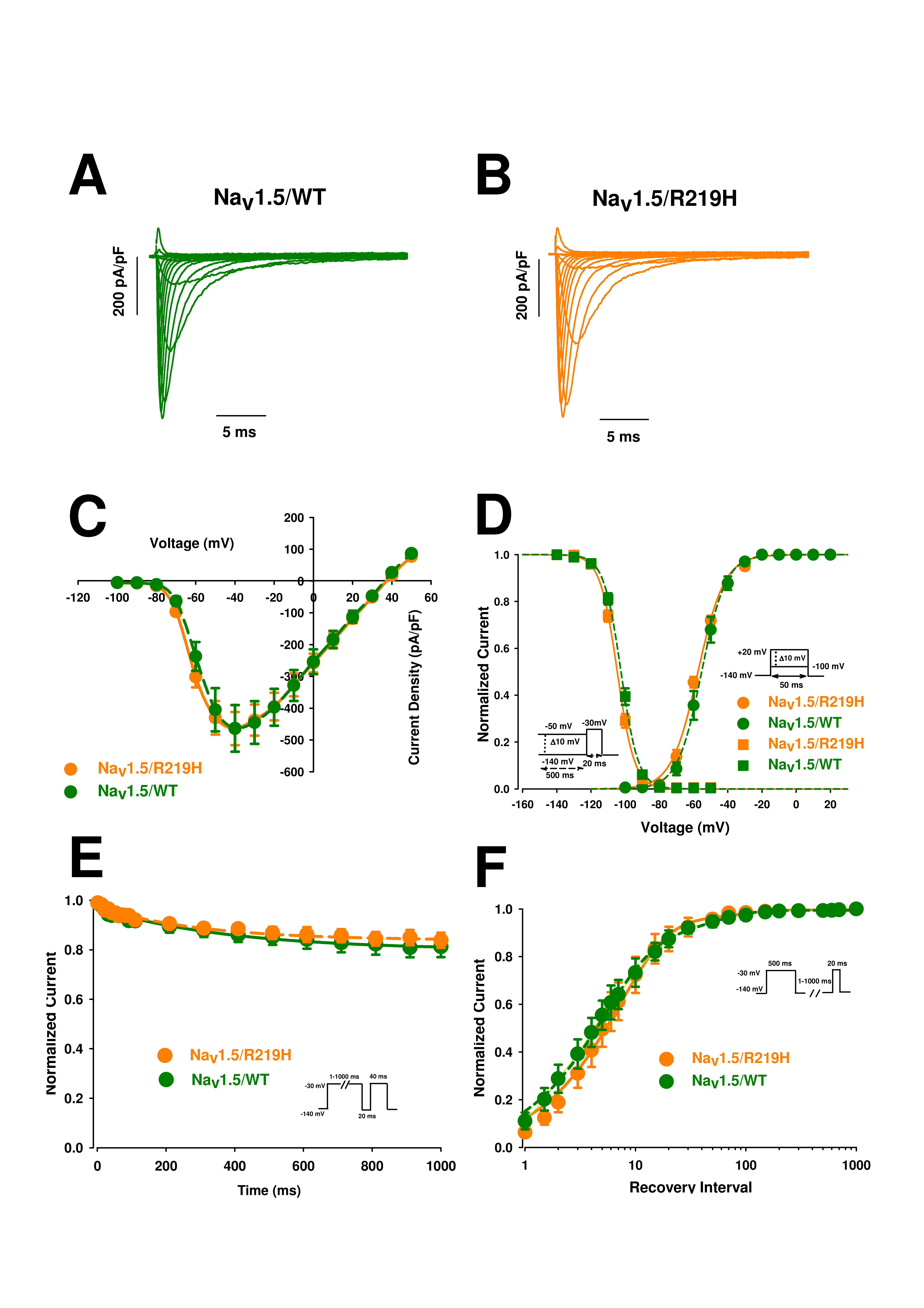

Supplement: Figure S4 — Biophysical characterization of Nav1.5/R219H Na+ channels expressed in tsA201 cells. Representative current traces recorded from Nav1.5/WT (a) and Nav1.5/R219H (b). Currents were elicited by depolarizing pulses starting at −100 mV to +50 mV with a 10 mV increment for each step from a holding potential of −140 mV, as shown in the inset protocol. (c) Current-voltage relationship of WT and R219H. Current amplitude was normalized to the membrane capacitance to generate the corresponding current density. (d) The activated and inactivated currents were generated from the protocols as inset. Using the same data as (c) and graphically determined reversal potentials (Erev), the Na+ conductance (G) for the various voltages was calculated from the equation G = I/(V−Erev). The fraction of the conductance was obtained by normalizing the various conductances at different voltages to the top values. Steady-state inactivation was measured by applying 500 ms pre-pulses ranging from −140 to −50 mV, followed by a 20 ms test pulse at −30 mV. The resulting data of steady-state activation and inactivation were fitted to a standard Boltzmann distribution. (e) Slow inactivation in WT and R219H. A two-pulse protocol as inset was used to generate the currents. The course of slow inactivation was assessed using a two-pulse protocol with an initial conditioning pre-pulse and a final test pulse. A −30 mV pre-pulse was applied at intervals varying from 1 to 1000 ms, followed by a step to −140 mV for 20 ms to allow the channels to recover from fast inactivation. The −30 mV test pulse was applied for 40 ms to estimate the fraction of channels available for activation. Time constants (shown in Table S2) were obtained using a mono-exponential function. (f) Time courses of recovery from slow inactivation in WT and R219H. A 500 ms conditioning pre-pulse was used to monitor recovery by a 20 ms test pulse after a variable recovery interval from 1 to 1000 ms (see protocol in inset). A two-exponential fun [file pone.0038331.s006.tif]

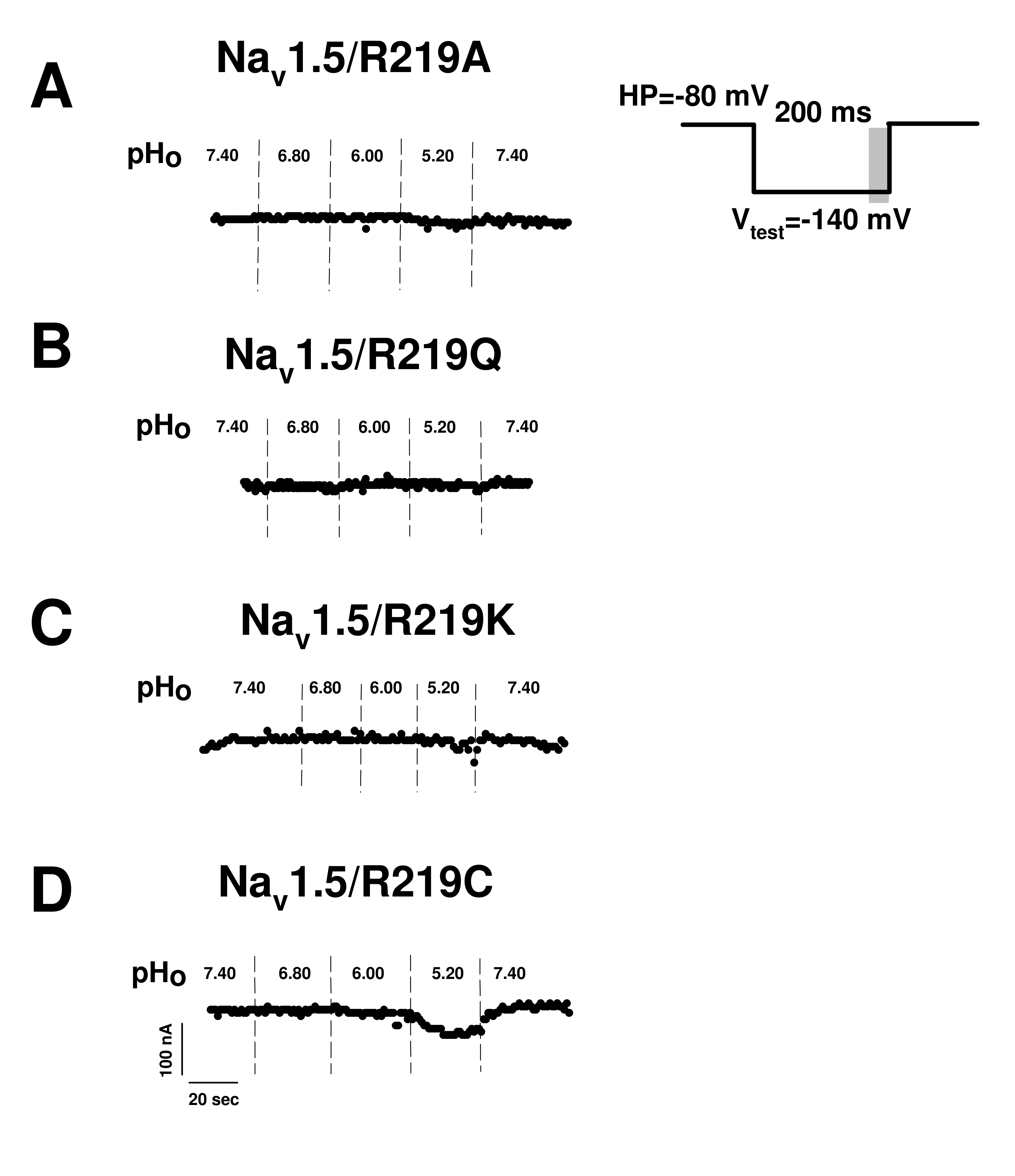

Supplement: Figure S5 — Effect of alanine, glutamine, lysine and cysteine substitution. The arginine 219 was substituted with alanine (a), glutamine (b), lysine (c) and cysteine (d), and oocytes expressing mutant channels were superfused with Na+-free NMDG solution at different pHo. Proton currents were measured every 2 seconds, using a hyperpolarizing pulse of −140 mV from a holding potential of −80 mV, as indicated in the inset. No proton currents could be seen in the presence of all mutant channels except for the cysteine mutant, where a slight inward deflection of the current at extreme acidic pHo value (5.20) (d) was observed, but we did not study this effect in greater detail. Similar results were obtained in two separate batches of oocytes. (TIF) [file pone.0038331.s007.tif]
